# ASSOCIATIONS BETWEEN NEIGHBORHOOD CHARACTERISTICS AND COGNITIVE FUNCTIONING TRAJECTORIES BY RACE/ETHNICITY

**DOI:** 10.1093/geroni/igac059.2075

**Published:** 2022-12-20

**Authors:** Amy Thierry, Heather Farmer

**Affiliations:** Xavier University of Louisiana, New Orleans, Louisiana, United States; University of Delaware, Newark, Delaware, United States

## Abstract

Older adults living in neighborhoods with high physical disorder and low social cohesion may be especially vulnerable to experiencing declining health. However, less is known about how neighborhoods may influence age-related patterns of cognitive functioning for different racial/ethnic groups. Therefore, we examine whether perceived neighborhood characteristics (safety, cleanliness, and social cohesion) are associated with trajectories of cognitive functioning across race/ethnicity. Using data from the 2006-2016 waves of the Health and Retirement Study, our study includes 11,870 non-Hispanic White, non-Hispanic Black, and Mexican adults 65 and older. We conducted linear mixed models stratified by race/ethnicity using an age-accelerated design to understand how neighborhood characteristics are related to trajectories of cognitive functioning (measured by the Telephone Interview for Cognitive Status; range: 0-35). We also explore if sociodemographic, behavioral, and health characteristics account for this association. Analyses revealed that cognitive functioning showed a quadratic change with age. Negative neighborhood perceptions were associated with declines in cognitive functioning with increasing age for each racial/ethnic group. Among White adults, all neighborhood characteristics were negatively associated with cognitive functioning in fully adjusted models. For Black adults, perceived uncleanliness was significantly associated with trajectories of cognitive functioning. In Mexican adults, perceptions of uncleanliness and lower cohesion were significantly associated with worse cognitive functioning, but not in fully adjusted models. These findings suggest that the perceived neighborhood context is an important social determinant of cognitive health for older adults, and distinct aspects of the neighborhood may be more correlated with cognitive functioning for certain racial/ethnic groups than others.

